# The evolution of C-peptide's role in diabetes care

**DOI:** 10.1097/MED.0000000000000947

**Published:** 2025-12-08

**Authors:** Laura Briggs, Alexander Read, Sarah Darch, Emma L. Williams, Wann Jia Loh, Julia S. Kenkre

**Affiliations:** a Department of Clinical Biochemistry, Imperial College Healthcare NHS Trust; b Department of Metabolism, Digestion and Reproduction, Imperial College, London, UK; c Department of Endocrinology, Changi General Hospital; d Duke-NUS Medical School, Singapore

**Keywords:** C-peptide, diabetes

## Abstract

**Purpose of review:**

Diabetes mellitus affects one in nine adults worldwide, with timely diagnosis and accurate classification being essential for patient management. C-peptide is an important biomarker in the diagnostic workup. As diabetes sub-typing and treatment options continue to evolve, this review will highlight the important aspects of C-peptide analysis and interpretation and additionally, evaluate its current and emerging clinical role.

**Recent findings:**

Several sample types and testing strategies such as fasting, random and stimulated C-peptide are available which are reviewed here. Random nonfasting C-peptide is convenient to perform in clinic and performs well compared to gold standard testing for classification of severe insulin deficiency and insulin dependence. C-peptide measurement may also be useful for classifying type 2 diabetes subtypes and in predicting response to treatment. Despite ongoing efforts towards standardization of C-peptide, variation still exists between analytical methods.

**Summary:**

This review summarizes recent literature relating to preanalytical, analytical and clinical aspects of C-peptide testing. Future research in this area may build on the role of C-peptide in predicting glycaemic control, clinical complications and response to pharmacotherapy.

## INTRODUCTION

The 31 amino acid C-peptide, a cleavage product of pro-insulin, is secreted into the portal vein from pancreatic β cells in an equimolar amount to insulin. In contrast to insulin, which is rapidly removed from the circulation via its receptor and subsequent lysosomal degradation, C-peptide is negligibly affected by hepatic first pass metabolism and thus has a three- to sevenfold longer circulating half-life [1]. Its assay does not commonly suffer from significant cross-reactivity sometimes observed when testing serum insulin in those on exogenous insulin analogues [2]. Furthermore, as it is renally excreted, it can be additionally tested in urine [3]. However, there are important testing considerations that clinicians should be aware of when interpreting individual patient results including: the impact of using different sample types such as serum versus plasma, blood versus urine, fasting versus stimulated samples along with considerations that cut-offs values and results may be affected by the assay used.

C-peptide measurement is of use clinically as a marker of β cell function, estimates of which are diagnostically and prognostically important in diabetes, and can also be used to calculate insulin secretion rates [4,5]. Results have been found to correlate with type and duration of diabetes, age of diagnosis and, in some clinical circumstances, treatment response [6,7]. A clear role of C-peptide in clinical practice is its discriminatory ability in confirming severe insulin deficiency states in insulin-dependent disease; not limited to type 1 diabetes (T1D) but also includes post-pancreatectomy and other secondary forms of insulin deficiency, and some monogenic forms of diabetes. However, there remains uncertainty over its role in clinical care of diabetes that is not completely insulin-dependent, particularly type 2 diabetes (T2D) [8]. This review will summarize the current evidence for measurement including assays and sample types as well as review the data on the clinical uses of C-peptide. 

**Box 1:**
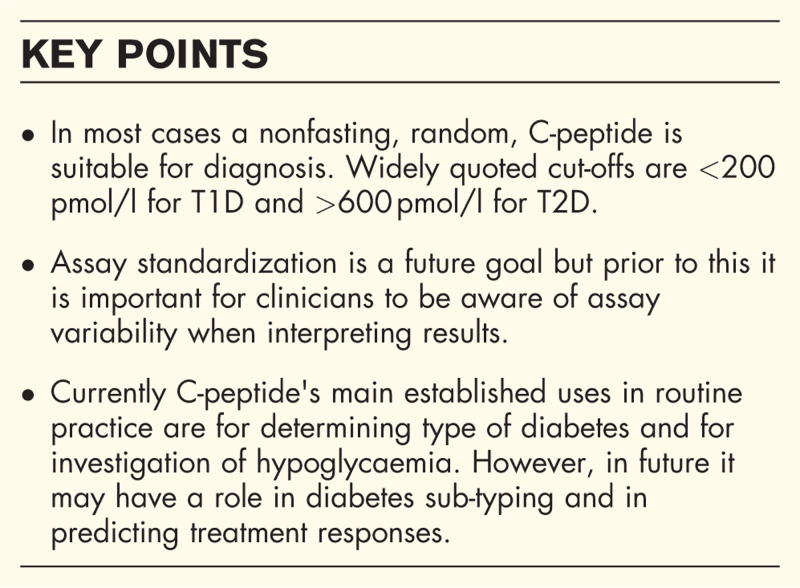
no caption available

## HOW SHOULD C-PEPTIDE BE MEASURED?

C-peptide can be measured in urine, either a spot urine, as a ratio with creatinine or 24 h collection, in the plasma and serum of blood or in capillary blood, see Table 1. Collection tube type, separation delays and storage of samples for C-peptide can all influence results and minimum requirements should be clearly communicated by the laboratory to clinical users.

**Table 1:** Methods of C-peptide measurement

Sample	Test	Clinical uses	Sample considerations	Advantages	Disadvantages
Urine	-Spot urinary C-peptide (UCP) -Urine C-peptide creatinine ratio (UCPCR) [9]	Mainly used in patients on insulin to assess endogenous insulin secretion [10]. Urine C-peptide has been utilised in decision models for identifying monogenic diabetes in adult and paediatric populations [11,12].	Stable for 3 days in Boric acid containers at ambient temperature or without preservative for 24 h. For long term storage, freeze. If stimulated measure required: collect postprandial sample taken approximately two hours after a carbohydrate- containing meal	Noninvasive, can be performed in an outpatient setting or postal testing. The absence of proteases found in blood, mean that C-peptide is more stable in urine. Useful where easy access to lab infrastructure isn’t available. Correlates with insulin deficiency in T2D	Its role in patients not on insulin treatment is limited. Inaccurate in Chronic Kidney Disease (CKD). UCPCR affected by gender and muscle mass as a result of differences in creatinine concentration. UCP alone is less sensitive than when expressed as ratio to creatinine.
	24 hr Urinary C-peptide collection (24 hr UCP) [13,14]	See above	See above	Useful in detecting insulin deficiency	Less convenient, time-consuming, requires good patient compliance. Inaccurate in CKD. Rarely used now as superseded by spot urine testing in most cases.
Capillary blood [15]	Point of Care (POC) finger prick testing	Potential uses as per laboratory blood C-peptide testing below. However, further validation required.	Capillary blood sample collected via POC finger prick	Minimally invasive, fast, simple to use POC test and convenient. Can be performed where there is limited access to lab infrastructure.	May be more costly than routine laboratory testing. Limited number of assays currently available.
Blood	Serum	See uses for random, fasting and stimulated samples below	Whole blood at room temperature stable for 6 h. Store frozen. C-peptide is stable for up to six freeze-thaw cycles in both serum and plasma [16]. Collection requirements for insulin may be more stringent than for C-peptidewhich may be an important consideration where samples are shared for both tests [16–18].	Less time consuming than urine sample	Invasive Needs to be separated within 6 h
	Plasma	See uses for random, fasting and stimulated samples below	EDTA whole blood at room temperature stable for at least 24 h. Store separated and frozen.	More stable prior to sample separation than serum samples	Invasive
	Fasting (after 8–10hr fast)	-In HOMA calculations in combination with glucose (e.g. HOMA-B). -To differentiate T1D versus T2D (especially in newly diagnosed, insulin-naïve patients) -To assess β-cell function in T2D (before escalating to insulin, to assess response to therapies over time -Screening for LADA (paired with autoantibodies)	See above	Less affected by confounders Simple, quick, correlates with diabetes type	Patients required to fast for 8–10hr Insufficient to detect subtle rises in C-peptide
	Random (nonfasting)	-Estimate of β-cell function (especially if fasting not feasible (e.g. children, elderly, acutely ill)) -To investigate hypoglycaemic episode (during or close to the episode and paired with glucose, insulin) -T1D versus T2D differentiation (require glucose concentration) -Insulinoma diagnosis (requires insulin and glucose concentrations)	See above	Easy to perform, quick, simple, correlates with diagnosis	Results dependent on time since last meal and composition of that meal
Stimulated C-peptide	Glucagon Stimulation test (GST) *(C-peptide measured up to 6 min after the intravenous administration of glucagon 1 mg)*	The standardised stimulus and sample timings, can be helpful with borderline fasting or random C-peptide results, or if discordance between clinical and biochemical phenotype of diabetes		Quick, sensitive, specific, reproducible, correlates with diagnosis	Performed at hospital More time consuming Nausea
	Mixed meal tolerance test (MMT) *(C-peptide is measured at regular intervals up to 120 min after the oral ingestion a mixed meal test)*	As above		Better tolerated than GST. Sensitive, specific, reproducible, correlates with diagnosis [19].	Time consuming, liquid mixed meals not widely available
	Oral Glucose Tolerance Test (OGTT) *(C-peptide is measured at regular intervals up to 120 min after the oral ingestion of 75 g of glucose)*	As above		Practical if at time of diagnostic OGTT.	Time consuming, limited evidence of predicting β cell function

### Fasting, random or stimulated C-peptide?

Differences in cut-off values and interpretation (see Table 2) primarily stem from the physiological differences between fasting, random or stimulated C-peptide [23]. Fasting C-peptide is the expression of steady state, that is, static response of β cell to ambient (arterial) glucose concentration, whereas random (nonfasting) C-peptide is primarily affected by the incretin effect and elevation in plasma glucose following ingestion of the previous meal [8].

**Table 2:** Examples of diagnostic cut-offs used for C-peptide in different sample types

Diabetes subtype	Fasting C-peptide cut-off (pmol/l)	Cut-off used in stimulated testing or for a random C-peptide within 5 h of eating (pmol/l)	UCPCR post home meal (nmol/mol)
Type 1 [20^▪^,^21^,^22^]	≤80	≤200	<0.2
Type 2 [20^▪^]	>600	>600	>0.6
Indeterminate: T1D/T2D/monogenic diabetes [20^▪^]		200-600	

The use of formal stimulation tests is currently proposed to be the gold standard for the primary outcome in the clinical investigation of insulin secretion, due to excellent sensitivity in detecting residual insulin secretion and for evaluation of the dynamics of β-cell response to provocative stimuli [24]. C-peptide is either measured up to 6 min after the intravenous administration of 1 mg of glucagon (GST), or at regular intervals up to 120 min after the oral ingestion of a MMT or 75 g glucose (OGTT). GST has been shown to be superior in sensitivity to use of glucose or tolbutamide as substrates, with a two-fold higher mean increase in C-peptide response [25]. Glucagon stimulates insulin secretion acutely and pharmacologically, producing a short-lived response. Conversely, the glucose load and the mixed meal stimulate insulin secretion physiologically, both in a glucose-dependent and a glucose-independent manner, producing a sustained response of insulin over time [26]. Therefore, although more time consuming, the MMT may better mimic the pancreatic response to ingestion of a meal during everyday life and has been shown to be more reproducible and better tolerated than the GST. Greenbaum *et al.* showed that the MMT is preferred for the assessment of β-cell function in therapeutic trials in type 1 diabetes [19].

Other C-peptide measures such as fasting blood C-peptide, or a postmeal UCPCR, give a reasonable approximation to the gold-standard, and high sensitivity and specificity in classifying diabetes. However, for routine clinical care, the most practical test would be a spot “random” nonfasting sample, collected when a patient is seen in a clinic setting. Berger *et al.* showed that random nonfasting C-peptide is more powerful than an overnight fasting sample and after glucagon stimulation in distinguishing T1D from T2D and can therefore be recommended as a classification tool, particularly in outpatients [27]. Hope *et al.* also showed that random nonfasting C-peptide measurement, in participants with insulin-treated diabetes, is strongly correlated with MMT C-peptide. Using a cut-off of <200 pmol/l, (measured by Roche immunoassay), random C-peptide was highly sensitive and specific for classifying severe insulin deficiency. Random nonfasting C-peptide could also identify individuals with T1D or insulin dependence, defined in the study as <600 pmol/l. This study also found that UCPCR correlated well with these cut-offs using thresholds of 0.2 and 0.6 nmol/mol [22].

In summary, in the majority of clinical scenarios, a simple test such as nonfasting random C-peptide, fasting C-peptide or postmeal UCPCR will be sufficient. If maximum accuracy is required, a stimulation test will be required, the MMT is best tolerated and has highest reproducibility, but is more time-consuming than the GST.

## METHODS OF ANALYSIS

C-peptide is routinely measured using commercial immunoassays on automated analysers. However, there remains significant variability between assays. Hörber *et al.* recently confirmed findings of two previous studies when comparing five widely used C-peptide immunoassays on different analysers. The analytical performance of the individual assays was satisfactory, but despite all the immunoassays being traceable to the international reference reagent (NIBSC code: 84/510), C-peptide measurements showed significant differences between analysers across the entire concentration range. The mean bias was largest (36.6%) between the immunoassays by Roche and Siemens Healthineers (ADVIA Centaur XPT): two samples giving results of 255 and 100 pmol/l on Roche assay, would give a result of 162 pmol/l and 63 pmol/l, respectively on the Siemens assay, which could have important implications for the interpretation of insulin secretion. Both Roche and Siemens Healthineers (ADVIA Centaur XPT) assays revealed large discrepancies compared to immunoassays by Abbott, DiaSorin, and Siemens Healthineers (Immulite 2000 XPi) which gave similar C-peptide results [28,29]. These methodological differences should be taken into consideration when defining clinically relevant cut-offs.

Harmonization can be achieved by calibration of all measurement procedures so that they are traceable to the same reference system. Reference methods have been established in the United States and Japan [30,31] and certified reference materials can ensure the comparability of results between these reference methods allowing for assignment of values to secondary reference materials [32]. It has been shown that recalibration by manufacturers using these matrix appropriate secondary reference materials (frozen serum samples with values assigned by the reference method) greatly improves the comparability of results among methods [30,31]. The majority of studies do not specify which C-peptide assay is used (54% of the 515 publications) and few provide information on detection limit, measurement range and variation. This lack of standardization makes it difficult to compare different studies or reproduce their results. For the clinician, it is important to recognize the variability between different C-peptide assays and take these differences into account when formulating thresholds [33]. There is an urgent need to implement and finalize the standardization of C-peptide measurements, to enable accurate and comparable results to facilitate the definition of universal reference values and clinical decision limits [34].

## C-PEPTIDE MEASUREMENT CONSIDERATIONS AND LIMITATIONS

Several biological and analytical factors can impact C-peptide concentration and its interpretation. Clearly, blood glucose concentration at the time of sampling will impact C-peptide concentration: hypoglycaemia reduces, while hyperglycaemia stimulates secretion of pro-insulin [35,36]. Additionally, international guidelines would suggest not testing C-peptide within two weeks of a significant hypoglycaemic episode when it is used for diagnostic classification of diabetes [37].

Furthermore, impaired renal clearance can increase measured serum C-peptide concentrations and thus concentrations should be interpreted with caution in individuals with chronic kidney disease [8]. Although eGFR cut-offs have not been established for their effect on C-peptide, contemporaneous measurement of blood glucose with C-peptide and creatinine is required to enable accurate interpretation. Furthermore, diagnostic C-peptide cut-offs (Table 2) are not validated in those with end stage renal disease.

C-peptide is most commonly measured by two-site immunoassay methodology which has some inherent limitations associated with it such as antibody-mediated interference [38]. Poly-reactive heterophilic antibodies and human antianimal antibodies can interfere in most two-site immunoassay methods [38]. One particularly important interference for C-peptide is the presence of antiinsulin auto-antibodies. Insulin autoimmune syndrome (IAS) is a rare but clinically important condition in which antibodies against insulin give rise to hyperinsulinaemic hypoglycaemia. In these patients, overestimation of C-peptide measured by immunoassay compared with liquid chromatography tandem mass spectrometry has been documented. The specific cause of the interference is not fully understood but may be due to insulin precursor or antibound proinsulin cross-reactivity [39,40]. Although IAS is uncommon, the immunoassay cross-reactivity for both insulin and C-peptide methods can give rise to potentially misleading results. Laboratory specialists and clinicians should be aware of the analytical limitations of C-peptide and the potential impact on interpretation.

## CLINICAL APPLICATIONS OF C-PEPTIDE

**FIGURE 1:**
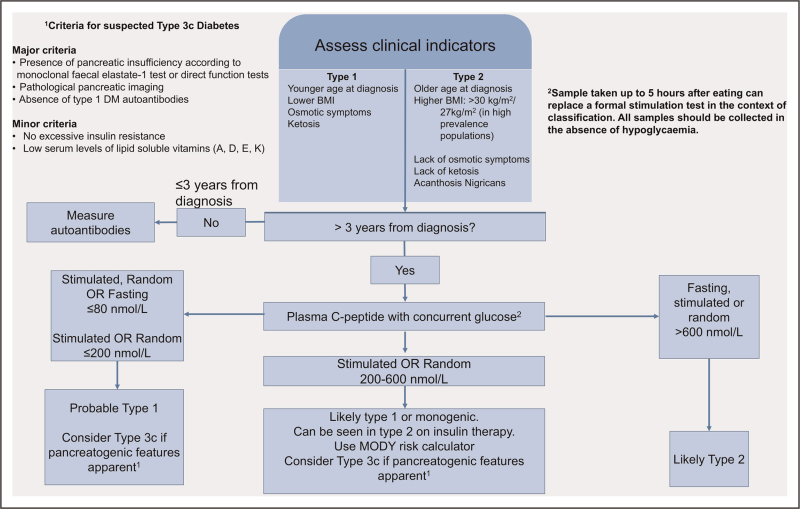
Schematic to show the role of C-peptide in current guidelines [23,22,36].

The primary uses of C-peptide are: determination of β cell secretory reserve including in models of secretion such as HOMA-% B [41], in international guidelines for diabetes sub-typing (see Fig. 1), as a measure of response to treatment, and prognostication of disease course [20^▪^,^37^].

### The use of C-peptide in diagnosis

Since the clinical presentation of patients with T1D, T2D and monogenic diabetes may overlap, C-peptide is a key tool to help differentiate them, see Fig. 1. At diagnosis, clinical indicators typically associated with T2D such as higher BMI, greater age, lack of weight loss, polyuria and polydipsia, lower HbA1c at diagnosis, lower diagnosis glucose, lack of family history of diabetes, and lack of ketoacidosis show increasing overlap with T1D. Whilst T1D often includes a ‘honeymoon period’ where β cell function is preserved but leads to complete insulin deficiency in 3–7 years following diagnosis, T2D can lead to decreased insulin secretory capacity dependent on disease course over similar time frames and dependent on treatment efficacy [42].

C-peptide is being recognised as a cost-effective intervention for patients with longer-standing diabetes (>3 years post-diagnosis). A study in Scotland used C-peptide as an indicator of retained secretory capacity where T1D had been diagnosed initially, enabling re-categorization to T2D or monogenic diabetes, discontinuation of insulin therapy and provision of more appropriate antidiabetic therapy [36]. Use of C-peptide in predictive models, or alongside HbA1c and OGTT may help identify T1DM patients which with a higher risk of progression to symptomatic disease and insulin dependence, but these should be verified in local populations with an assessment of the cost of testing versus risk of escalating morbidity and potential cost in the absence of earlier intervention [43]. Moreover, the use of C-peptide in more complex predictive models, as suggested by a recent study of T1D predictive models in antibody-positive patients, must be balanced with the time and cost of acquiring such additional variables [44].

Low and undetectable plasma C-peptide provide strong evidence of a diagnosis of T1D, however, interpretation is complicated by the association of earlier age of diagnosis of T1D with lower C-peptide concentrations, as suggested in studies focused on diagnosis of diabetes in paediatric populations [45]. Interpretation is further complicated by the increases in C-peptide seen in obesity and accompanying insulin resistance, now more prevalent amongst the population with T1D than seen previously.

Studies on staging those who went on to develop T1D (recruited from relatives and presence of at least 1 autoantibody) suggest about a third have C-peptide ≤200 pmol/l by 24 months [46]. In conjunction with positive autoantibodies this suggests autoimmune destruction in T1D. C-peptide *<*50 nmol/l is almost always associated with T1D. Conversely, fasting C-peptide *>*600 pmol/l, as an indicator of insulin secretion, has been used to suggest T2D with the absence of insulin requirement within 3 years [47]. A recent meta-analysis suggest low C-peptide is diagnostic of T1D across different heterogeneous ancestry reflected by replication of results in middle east and north African regions [48,49].

C-peptide has been used with other phenotypic and/or biochemical data to sub-categorize patients with T2D into clusters, each with varying rates of disease progression and complications [50,51]. Clustering has been reproduced amongst populations across geographies and of different ancestry and has been shown to be predictive of outcomes measures at 5-year follow up [52–54]. Bancks *et al.* showed, in a sub-population of T2D retrospectively classified as ‘poor glucose control’, when given intensive lifestyle intervention as primary treatment, they had increased risk of poor cardiovascular outcomes [55]. However, there remains a lack of data to support that cluster-driven management is cost-effective and leads to improved outcomes above and beyond the use of current clinical phenotyping.

Finally, Type 3c diabetes, related to pancreatic dysfunction, encompassing diabetes caused by chronic pancreatitis and rarer causes such as pancreatic cancer, cystic fibrosis, surgery and haemochromatosis [56], is under diagnosed with an estimated prevalence of 1–9% of those living with diabetes. Diagnostic criteria include use of C-peptide as minor criterion [57].

### C-Peptide for predicting treatment response

#### Predicting diabetes remission and/or future treatment discontinuation

Diabetes remission, normoglycaemia without requirement for hypoglycaemic agents, can be seen following weight loss, induced by both dietary interventions and bariatric surgery. Higher C-peptide as a marker of residual β-cell function, is associated with increased likelihood of remission following bariatric surgery, although there is large variation in suggested cut-offs to determine this [58]. Prior to dietary intervention, fasting C-peptide was not predictive in the DIRECT trial, although this only enrolled patients with a limited duration of T2D (<6 years), [59,60]. Finally, in ketosis-prone diabetes, C-peptide may predict ability to discontinue insulin following diabetic ketoacidosis (DKA) [61–63].

#### Risk of hypoglycaemia

Sustained insulin secretory capacity, defined in the DCCT by a stimulated C-peptide >200 pmol/l at baseline and at least 1 year later, is also associated with lower rates of hypoglycaemia in those treated with intensive insulin therapy [64]. Random or stimulated C-peptide concentrations <200 pmol/l are predictive of increased coefficient of variation (CV) in continuous glucose monitoring (CGM); conversely, those with C-peptide >600 pmol/l have an associated CV ≤36% [65]. In T2D treated with insulin, random, nonfasting, C-peptide is associated with increased glucose variability and [66] hypoglycaemia. In T1D, following islet transplant, stimulated C-peptide (MMT) <200 pmol/l was indicative of increased risk of hypoglycaemia as defined by glucose <3 mmol/l on CGM [67]. Recent evidence has sought to identify a lower cut-off of ~15 pmol/l or below for random C-peptide to indicate increased % time below range and therefore higher risk of hypoglycaemia in type 1 diabetes patients [68^▪^]. However, weak diagnostic performance, lack of assay harmonization and imprecision near the limit of quantitation limit the value of this cut-off [66].

#### Glycaemic control and microvascular complications

Whilst there are studies where lower fasting C-peptide concentrations were associated with increased prevalence of diabetic neuropathy and retinopathy, overall, our literature search suggests that there is insufficient evidence to support the use of C-peptide in assessing glycaemic control and microvascular complications in T1D or T2D [69,70].

#### Response to pharmacotherapy

There is limited evidence that lower C-peptide could be used to predict how soon insulin therapy may be required in those with T2D and one study suggesting it could be used to guide insulin dosage in those on an insulin pump [71]. Perhaps, of greater clinical use and with more robust evidence, is its potential role in guiding withdrawal of insulin [72].

C-peptide may also have a future additional use in predicting treatment response to newer agents. GLP-1 analogues improve β cell compensation for insulin resistance. Jones *et al.* previously showed, as would be expected, that those with a low secretory reserve, as defined by a C-peptide <250 pmol/l, have a significantly reduced glycaemic response to GLP-1 analogues [73]. Moreover, however, using C-peptide area under the curve as an end-point, Dejgaard *et al.* observed a significant difference between liraglutide and placebo in patients with newly diagnosed T1D. Liraglutide has been shown to preserve C-peptide AUC leading to fewer episodes of hypoglycaemia and increased time without need for insulin treatment [74]. The preserved C-peptide concentrations in this case represent a proxy for secretory capacity that may demonstrate treatment efficacy. Another recent trial assessing semaglutide plus metformin versus metformin alone in T2D, used the increased fasting and postprandial C-peptide seen in the semaglutide group, as a marker of treatment efficacy [75].

C-peptide may further help to identify patients at higher risk of euglycaemic diabetic ketoacidosis before initiating SGLT2 inhibitors, with lower C-peptide concentrations being associated with increased risk [76–78].

In summary, there is emerging evidence to support C-peptide is guiding insulin withdrawal, as a proxy of secretory capacity that is altered by successful treatment with GLP1-R analogues and potentially a future role for predicting DKA in those commencing SGLT2 inhibitors.

## CONCLUSION

This review highlights the impact of preanalytical factors and analytical methodological differences upon C-peptide measurement, which need to be considered when interpreting results. C-peptide testing is available for a variety of sample types, but a random plasma or serum C-peptide sample offers key advantages, including patient convenience and correlates well with stimulated C-peptide testing. Used alongside clinical assessment and other laboratory tests, C-peptide is a key biomarker for the classification of diabetes. Although C-peptide measurement is primarily utilised in the determination of β-cell secretory reserve, it may also be a useful marker in predicting treatment response.

## Acknowledgements

*None.*

### Financial support and sponsorship

*None.*

### Conflicts of interest

*There are no conflicts of interest.*

## REFERENCES AND RECOMMENDED READING

Papers of particular interest, published within the annual period of review, have been highlighted as:

▪ of special interest
▪▪ of outstanding interest
